# Supplementary material for: Dynalign II: common secondary structure prediction for RNA homologs with domain insertions
Source: Nucleic Acids Res. 2014 Nov 21;42(22):13939–48. doi: 10.1093/nar/gku1172 (PMC4267632; doi:10.1093/nar/gku1172)
Supplement: SUPPLEMENTARY DATA [file supp_gku1172_nar-02021-z-2014-File012.zip › manual/Text/multilign.html]

RNAstructure Command Line Help: multilign


|  |  |  |
| --- | --- | --- |
|  | RNAstructure Command Line Help multilign | - Contents - Index |
| multilign is used to find the lowest free energy common secondary structure for more than two homologous sequences. It uses multiple iterations of Dynalign to predict the conserved structure. It has two distinct executables, a serial program called multilign and a parallelized program called multilign-smp for use in shared memory environments. Note that at least three sequences are necessary for multilign to run. Note that on Macintosh OS X, and some versions of Linux, there is a small default stack limit. To run multilign, the stack limit needs to be increased. On the default shell, bash, use *ulimit -s 4096*. If you are using tcsh, use *limit stack 4096*. The limits can be increased as necessary if the given values above are not sufficient. USAGE 1: multilign <configuration file> USAGE 2: multilign-smp <configuration file> Required parameters: <configuration file> \| The name of a file containing required configuration data. Options which don't require added values: NONE Options which require added values: NONE Configuration file format: The following is a description of valid options allowed in the configuration file. The example is based on multilign\_sample.conf, a standard example found in the examples directory of the RNAstructure repository. ``` ################################################################ # IMPORTANT CONFIG FILE FORMAT NOTES: # # Config file options described below are not case sensitive. # # Option lines may be specified by the option name followed by an equals sign and the option's desired value. # When specifying an option, there may be nothing else on the line. # If an option is specified more than once, the last specification is used. # <option> = <value> # # Specifying comment lines: # Comment lines must begin with "#" followed by a space. # There may not be more than one "#" in a comment line. # However, a comment line may be an unbroken string of "#", as in a divider between sets of options. # # Blank lines are skipped. # Any leading or trailing whitespace is ignored. # Variables may not contain internal whitespace. # # Syntax errors produce a warning to standard output and are then ignored. ################################################################ ################################################################ # Input options. # If an appropriate group of these values is not defined, the program will exit. ################################################################ # There are two separate ways to specify a group of input sequences: # 1. Place sequence file names in brackets separated by semicolons. # Note that there cannot be any spaces whatsoever between the brackets. # The list must hold "SequenceNumber" sequences. # 2. Each successive sequence "n" from 1 to SequenceNumber is specified as "Seq<n>." # Note that there cannot be any spaces in the file name. InSeq = {seq1;seq2;seq3;} Seq<n> = <seq file n> # There are two separate ways to specify a group of output CT files: # 1. Place CT file names in brackets separated by semicolons. # Note that there cannot be any spaces whatsoever between the brackets. # The list must hold "SequenceNumber" CT files. # 2. Each successive CT file "n" from 1 to SequenceNumber is specified as "CT<n>." # Note that there cannot be any spaces in the file name. OutCT = {ct1;ct2;ct3;} CT<n> = <ct file n> # Alignment specifies the output filename for the multiple alignment. Alignment = <alignment file name> # SequenceNumber specifies the number of sequences given for calculation. # This is only needed if both sequences and CT files are specified individually. SequenceNumber = <number of sequences> # Note that sequence file, CT file, and sequence number options must appear in one of two valid combinations, depending if # files are specified singly or in a group. # # Singly: # Alignment = <alignment file name> # SequenceNumber = <n> # Seq1 = <seq file 1> # CT1 = <ct file 1> # ... repeat single entries of Seq and CT until <n> is reached # # Groups: # Alignment = <alignment file name> # InSeq = {seq1;seq2;seq3;} # OutCT = {ct1;ct2;ct3;} # Constraint<1> - Constraint<SequenceNumber> specifies the folding constraint file names for each successive sequence "n" # from 1 to SequenceNumber. # Note that there cannot be any spaces in the file name. # Folding constraint files are not required for any sequence. Constraint<n> = <constraint file n> # SHAPE<1> - SHAPE<SequenceNumber> specifies the SHAPE file names for each successive sequence "n" # from 1 to SequenceNumber. # SHAPE files are not required for any sequence. # Note that there cannot be any spaces in the file name. SHAPE<n> = <SHAPE file n> ################################################################ # Options with default values if not explicitly specified. # This section of defaults contains general options. ################################################################ # DNA specifies whether multilign will be run with RNA or DNA thermodynamics. # Use 0 to specify RNA thermodynamics, and 1 to specify DNA thermodynamics. # Its default value is 0, which specifies RNA. DNA = 0 # SHAPEintercept specifies the SHAPE intercept used by multilign. # Note that if specified, this value is only used if one or more SHAPE files is also specified. # Its default value is 1.8 kcal/mol. SHAPEintercept = 1.8 # SHAPEslope specifies the SHAPE slope used by multilign. # Note that if specified, this value is only used if one or more SHAPE files is also specified. # Its default value is -0.6 kcal/mol. SHAPEslope = -0.6 # Temperature specifies the temperature at which multilign is run, in Kelvin. # Its default value is 310.15 K, which is 37 degrees C. Temperature = 310.15 # Processors specifies the number of processors multilign is run on. # Note that this flag only has an effect when multilign-smp, the parallel version of multilign, is run. # Its default value is 1. Processors = 1 ################################################################ # Options with default values if not explicitly specified. # This section of defaults contains options unique to multilign. ################################################################ # IndexSeq specifies the sequence chosen to be the index sequence. # Its default value is 1. IndexSeq = 1 # Iterations specifies how many iterations to run. # Its default value is 2. Iterations = 2 # KeepIntermediate specifies if pairwise save files and alignments are deleted once calculations finish. # Use 0 to specify that files will be deleted, and 1 to specify that they will not be deleted. # Its default value is 0, which specifies that intermediate files will be deleted. # NOTE: On Windows, file locking prevents the removal of intermediate files. # On Windows, KeepIntermediate = 0 will result in a warning message that one or more files could not be deleted. # This warning message does not interfere with the operation of Multilign. KeepIntermediate = 0 # There are two different ways to specify maxdsvchange. # MaxDsvChange specifies the maximum interval above the lowest free energy for a base pair to be allowed. # Its default value is 1. MaxDsvChange = 1 maxdsvchange = 1 # MaxPairs specifies the maximum number of base pairs to be allowed. # Its default value is -1, which specifies the average length of all sequences. MaxPairs = -1 # Random specifies whether to randomize the sequence order. # Use 0 to specify no randomization, and 1 to specify randomization. # Its default value is 0, which specifies no randomization. Random = 0 ################################################################ # Options with default values if not explicitly specified. # This section of defaults contains options unique to Dynalign. ################################################################ # Gap specifies the per nucleotide insert penalty for alignments. # Its default value is 0.4. Gap = 0.4 # Insert specifies whether single base pair inserts will be allowed. # Use 0 to specify no inserts, and 1 to specify inserts. # Its default value is 1, which specifies inserts. Insert = 1 # Local specifies whether local or global folding should be done. # Use 0 to specify global folding, and 1 to specify local folding. # Its default value is 0, which specifies global folding. Local = 0 # MaxPercent specifies the maximum percent energy difference. # Its default value is 20 (percent). MaxPercent = 20 # MaxPercentSingle specifies the maximum percent difference in folding free energy change from single sequence folding for pairs # that will be allowed in a subsequent calculation. # This is used to save calculation time by pre-screening allowed pairs. # Its default value is 30 (percent). MaxPercentSingle = 30 # MaxStructures specifies the maximum number of structures to calculate. # Its default value is 750 structures. MaxStructures = 750 # Separation specifies the traditional M parameter. # -99 indicates that the alignment constraint (preferred) method is used. # Its default value is -99. Separation = -99 # WindowAlign specifies the alignment window. # Its default value is 1. WindowAlign = 1 # WindowBP specifies the base pair window. # Its default value is 2. WindowBP = 2 						 ``` Notice: Intermediate files with suffixes of ".dsv" or ".aout" are written to the path of the CT file for the index sequence, during Multilign calculations. ".dsv" and ".aout" files are the Dynalign save files and alignment files, respectively, generated by progressive pairwise Dynalign calculations. The intermediate files are named as "j.i\_<seq1\_filename>\_<seq2\_filename>.dsv" and "j.i\_<seq1\_filename>\_<seq2\_filename>.aout", where j is the iteration ordinal of the Multilign calulation and i is the pairwise dynalign calculation ordinal of the current iteration. The intermediate files are placed in the same directory as the CT file associated with the index sequence. So, for example, if the index sequence was the third one (3), all the intermediate files would be placed in the same directory as the CT file written for sequence 3. References: 1. Xu, Z., and Mathews, D.H. "Multilign: An Algorithm to Predict Secondary Structures Conserved in Multiple RNA Sequences." Submitted. 2. Reuter, J.S. and Mathews, D.H. "RNAstructure: software for RNA secondary structure prediction and analysis." *BMC Bioinformatics*, 11:129. (2010). 3. Harmanci, A.O., Sharma, G. and Mathews, D.H. "Efficient Pairwise RNA Structure Prediction Using Probabilistic Alignment Constraints in Dynalign." *BMC Bioinformatics*, 8:130. (2007). 4. Uzilov, A.V., Keegan, J.M. and Mathews, D.H. "Detection of non-coding RNAs on the basis of predicted secondary structure formation free energy change." *BMC Bioinformatics*, 7:173. (2006). 5. Mathews, D.H. "Predicting a set of minimal free energy RNA secondary structures common to two sequences." *Bioinformatics*, 21:2246-2253. (2005). 6. Mathews, D.H. and Turner, D.H. "Dynalign: An algorithm for finding the secondary structure common to two RNA sequences." *J. Mol. Biol.*, 317:191-203. (2002). | | |
| Visit The Mathews Lab RNAstructure Page for updates and latest information. | | |
